# Supplementary material for: The Effect of Autogenic Training in a Form of Audio Recording on Sleep Quality and Physiological Stress Reactions of University Athletes—Pilot Study
Source: Int J Environ Res Public Health. 2022 Nov 30;19(23):16043. doi: 10.3390/ijerph192316043 (PMC9737934; doi:10.3390/ijerph192316043)
Supplement: Supplementary file 1 [file ijerph-19-16043-s001.zip › ijerph-1987525-supplementary.pdf]

Table S1. Comparison of PRE and POST measurements among EG and CG and between the groups.

Data Source	Parameter	EG (n=11)				CG (n=11)				EG/CG Diff			
		Median		Pre-Post diff		Median		Pre-Post diff		Pre-Pre		Post-Post	
		PRE	POST	Z [b]	p	PRE	POST	Z [b]	p	Z	p	Z [c]	p
Questionnaire	PSQI	6.00	5.00	2.52	0.011	6.00	5.00	1.63	0.103	−0.07	0.945	−0.97	0.330
Actigraphy	Sleep duration (min.)	428.00	402.50	0.53	0.594	394.00	399.17	0.66	0.508	0.45	0.653	0.37	0.713
ADS application	Sleep duration (min.)	498.50	532.75	2.13	0.033	509.75	481.50	0.89	0.374	−0.66	0.511	−1.08	0.279
	Sleep latency (min.)	15.63	13.75	0.54	0.593	11.75	11.25	0.49	0.624	−0.36	0.718	−0.79	0.429
	Sleep quality [a]	3.50	3.25	0.10	0.918	3.75	3.75	0.51	0.609	−0.10	0.920	−0.74	0.462
	Energy level [a]	3.50	3.25	0.43	0.666	3.00	3.25	1.13	0.261	−0.89	0.372	−0.34	0.738
	Stress level [a]	1.75	1.00	1.15	0.251	1.25	1.50	0.71	0.481	−1.77	0.077	−0.38	0.707
SC sensor [d]	Baseline	2.13	1.87	0.62	0.533	3.39	2.94	0.53	0.594	−0.72	0.470	−0.98	0.325
	Event	5.67	3.81	1.60	0.110	7.91	6.45	0.09	0.929	−0.53	0.599	−1.18	0.237
	Post-event	4.53	3.04	1.87	0.062	6.63	5.58	0.44	0.657	−0.85	0.393	−0.98	0.325
Temp sensor [d]	Baseline	33.48	32.65	0.53	0.594	31.92	33.90	0.09	0.929	−0.07	0.948	0.30	0.768
	Event	33.93	34.33	0.56	0.575	31.70	33.64	0.04	0.965	0.26	0.793	0.53	0.599
	Post-event	34.47	34.80	1.11	0.266	31.63	34.05	0.80	0.424	0.79	0.431	0.20	0.844
BVP sensor [d]	Baseline	36.50	36.51	0.27	0.655	36.47	36.48	0.31	0.755	0.69	0.491	1.08	0.279
	Event	36.36	36.39	0.46	0.646	36.38	36.39	0.36	0.655	0.13	0.896	0.69	0.491
	Post-event	36.39	36.38	0.67	0.562	36.40	36.36	1.27	0.238	−0.39	0.694	1.15	0.251
HR sensor [d]	Baseline	63.23	68.52	1.60	0.110	69.53	73.67	2.04	0.041	−0.66	0.511	−1.25	0.212
	Event	73.03	74.32	1.33	0.182	77.78	77.79	0.89	0.374	−0.85	0.393	−0.98	0.325
	Post-event	61.94	66.86	1.60	0.110	69.20	70.44	0.71	0.477	−1.05	0.293	−0.66	0.511
Resp sensor [d]	Baseline	14.64	14.32	0.36	0.722	13.82	13.58	0.62	0.534	0.20	0.844	0.20	0.844
	Event	13.55	13.41	0.27	0.790	13.20	13.15	1.16	0.266	0.39	0.694	0.79	0.431
	Post-event	14.67	14.80	0.22	0.824	13.30	12.89	1.42	0.155	0.13	0.896	0.72	0.470

Note: [a] subjective evaluation on a Likert scale from 1 to 5; [b] results of Wilcoxon test; [c] results of Mann-Whitney U test; [d] Measurements taken with the biofeedback device containing three 5-minutes’ stages: baseline (anticipation of stress), event (arithmetic task-induced stress) and post-event (post-activity rest).

Table S2. Mean and standard deviation values for PRE and POST measurements among EG and CG.

Data Source	Parameter	EG (*n* = 11)				CG (*n* = 11)			
		PRE		POST		PRE		POST	
		M ± SD	Range	M ± SD	Range	M ± SD	Range	M ± SD	Range
Questionnaire	PSQI	6.27 ± 1.19	5–8	4.64 ± 1.57	2–8	6.27 ± 1.01	5–8	5.27 ± 1.74	3–8
Actigraphy	Sleep duration (min.)	395.17 ± 78.92	250–473	403.7 ± 48.2	313–467	393.7 ± 57.31	292–477	383.02 ± 75.16	217–489
ADS application	Sleep duration (min.)	478.09 ± 65.18	370–566	516.82 ± 78.06	421–688	493.7 ± 79.06	324–594	468.07 ± 95.63	218–596
	Sleep latency (min.)	17.74 ± 9.7	4–34	18.21 ± 11.32	7–40	17.0 ± 10.98	8–40	15.23 ± 10.05	3–36
	Sleep quality [a]	3.57 ± 0.32	3.0–4.0	3.55 ± 0.46	3.0–4.25	3.61 ± 0.62	2.75–4.75	3.66 ± 0.64	2.25–4.75
	Energy level [a]	3.23 ± 0.69	2.0–4.25	3.3 ± 0.53	2.5–4.0	3.04 ± 0.87	2.0–5.0	3.26 ± 0.85	1.25–4.5
	Stress level [a]	1.9 ± 0.72	1.0–3.25	1.59 ± 0.81	1.0–3.25	1.36 ± 0.36	1.0–2.0	1.5 ± 0.47	1.0–2.5
SC sensor [b]	Baseline	2.18 ± 0.99	1.05–4.09	2.04 ± 1.33	0.41–4.98	2.97 ± 1.86	0.41–5.62	3.35 ± 2.57	0.50–9.48
	Event	6.08 ± 2.79	2.63–11.22	4.77 ± 2.7	1.29–9.85	7.21 ± 4.24	1.59–14.19	7.19 ± 4.53	1.52–14.6
	Post-event	5.26 ± 2.94	1.96–10.41	4.06 ± 2.78	0.97–9.44	6.7 ± 4.1	0.91–13.15	6.1 ± 4.11	1.19–13.62
Temp sensor [b]	Baseline	31.18 ± 4.27	24.30–35.48	32.06 ± 3.31	24.93–34.83	30.99 ± 4.04	24.95–34.94	31.25 ± 4.85	23.10–34.83
	Event	32.02 ± 3.72	24.89–35.42	32.85 ± 3.32	25.31–35.18	31.25 ± 4.03	24.11–35.29	31.72 ± 4.66	23.05–35.56
	Post-event	32.57 ± 3.28	25.86–35.47	33.1 ± 3.41	25.69–35.41	31.47 ± 3.68	24.14–35.39	32.25 ± 4.51	23.06–35.72
BVP sensor [b]	Baseline	36.5 ± 0.05	36.40–36.58	36.5 ± 0.07	36.34–36.57	36.49 ± 0.07	36.4–36.6	36.48 ± 0.04	36.44–36.55
	Event	36.38 ± 0.07	36.23–36.47	36.39 ± 0.05	36.26–36.45	36.36 ± 0.06	36.26–36.43	36.37 ± 0.06	36.26–36.42
	Post-event	36.38 ± 0.04	36.28–36.43	36.4 ± 0.06	36.33–36.5	36.4 ± 0.05	36.33–36.51	36.38 ± 0.07	36.32–36.57
HR sensor [b]	Baseline	64.2 ± 11.31	43.35–80.68	67.17 ± 10.3	50.12–82.94	67.13 ± 6.9	52.66–73.95	74.86 ± 13.22	51.37–95.94
	Event	73.83 ± 13.82	49.57–97.1	74.13 ± 12.24	53.56–91.6	79.8 ± 11.45	59.24–100.17	82.59 ± 13.84	64.32–101.6
	Post-event	62.84 ± 10.72	44.44–81.13	65.84 ± 9.75	50.28–80.27	67.51 ± 8.67	52.48–79.05	69.68 ± 16.06	40.73–90.25
Resp sensor [b]	Baseline	13.34 ± 3.42	7.75–18.02	13.52 ± 2.92	9.44–18.41	13.47 ± 2.46	9.4–17.59	13.68 ± 2.92	8.59–18.29
	Event	13.75 ± 1.11	11.71–15.31	13.78 ± 1.09	12.62–16.23	13.58 ± 1.13	12.09–15.49	13.34 ± 1.05	11.88–15.19
	Post-event	14.13 ± 2.78	8.75–18.57	13.98 ± 2.79	8.09–18.19	14.06 ± 2.49	9.84–17.75	13.38 ± 2.47	9.70–18.0

Note: [a] subjective evaluation on a Likert scale from 1 to 5; [b] Measurements taken with the biofeedback device containing three 5-minutes' stages: baseline (anticipation of stress), event (arithmetic task-induced stress) and post-event (post-activity rest).
